# Tailoring dissemination strategies to increase evidence-informed policymaking for opioid use disorder treatment: study protocol

**DOI:** 10.1186/s43058-023-00396-5

**Published:** 2023-02-16

**Authors:** Erika L. Crable, Colleen M. Grogan, Jonathan Purtle, Scott C. Roesch, Gregory A. Aarons

**Affiliations:** 1 Department of Psychiatry, University of California, San Diego, La Jolla, CA USA; 2 Child and Adolescent Services Research Center, San Diego, CA USA; 3 University of California, San Diego Altman Clinical and Translational Research Institute Dissemination and Implementation Science Center, La Jolla, CA USA; 4 Crown Family School of Social Work, Policy, and Practice, The University of Chicago, Chicago, IL USA; 5 Department of Public Health Policy and Management, New York University School of Global Public Health, New York City, NY USA; 6 Global Center for Implementation Science, New York University School of Global Public Health, New York City, NY USA; 7 Department of Psychology, San Diego State University, San Diego, CA USA

**Keywords:** Dissemination strategies, EPIS framework, Information dissemination, Managed care populations, Medicaid, Opioid-related disorders, Policy, Politics, Prior authorization, Substance use disorder treatment

## Abstract

**Background:**

Policy is a powerful tool for systematically altering healthcare access and quality, but the research to policy gap impedes translating evidence-based practices into public policy and limits widespread improvements in service and population health outcomes. The US opioid epidemic disproportionately impacts Medicaid members who rely on publicly funded benefits to access evidence-based treatment including medications for opioid use disorder (MOUD). A myriad of misaligned policies and evidence-use behaviors by policymakers across federal agencies, state Medicaid agencies, and managed care organizations limit coverage of and access to MOUD for Medicaid members. Dissemination strategies that improve policymakers’ use of current evidence are critical to improving MOUD benefits and reducing health disparities. However, no research describes key determinants of Medicaid policymakers’ evidence use behaviors or preferences, and few studies have examined data-driven approaches to developing dissemination strategies to enhance evidence-informed policymaking. This study aims to identify determinants and intermediaries that influence policymakers’ evidence use behaviors, then develop and test data-driven tailored dissemination strategies that promote MOUD coverage in benefit arrays.

**Methods:**

Guided by the Exploration, Preparation, Implementation, and Sustainment (EPIS) framework, we will conduct a national survey of state Medicaid agency and managed care organization policymakers to identify determinants and intermediaries that influence how they seek, receive, and use research in their decision-making processes. We will use latent class methods to empirically identify subgroups of agencies with distinct evidence use behaviors. A 10-step dissemination strategy development and specification process will be used to tailor strategies to significant predictors identified for each latent class. Tailored dissemination strategies will be deployed to each class of policymakers and assessed for their acceptability, appropriateness, and feasibility for delivering evidence about MOUD benefit design.

**Discussion:**

This study will illuminate key determinants and intermediaries that influence policymakers’ evidence use behaviors when designing benefits for MOUD. This study will produce a critically needed set of data-driven, tailored policy dissemination strategies. Study results will inform a subsequent multi-site trial measuring the effectiveness of tailored dissemination strategies on MOUD benefit design and implementation. Lessons from dissemination strategy development will inform future research about policymakers’ evidence use preferences and offer a replicable process for tailoring dissemination strategies.

**Supplementary Information:**

The online version contains supplementary material available at 10.1186/s43058-023-00396-5.

Contributions to the literature

- This study will advance our knowledge of data-driven methods for developing and testing dissemination strategies to enhance policymakers’ use of research evidence.
- This is one of the first studies to examine policymakers’ perceived utility of tailored dissemination strategies.
- This is the first funded study examining dissemination strategy development and use for public insurance, and findings will advance efforts to improve access to evidence-based substance use treatment.
- This study will highlight evidence-informed decision-making behaviors across Medicaid and Children’s Health Insurance Program benefit arrays nationwide, including understudied programs in US territories.

## Background

Policy is a powerful tool for systematically altering access and quality of healthcare services. But the “research to policy gap” represents a significant impediment to translating scientific knowledge about evidence-based practices (EBPs) into public policy, limiting widespread improvements in service and population health outcomes [1]. Common barriers to translating research into health policy include weak relationships between the producers and users of research, poor alignment between research questions and policymaker priorities, and untimely and inadequate dissemination of research in accessible language and formats [1–4]. Complex political contexts where partisan ideologies, advocacy interests, and budgetary and resource constraints interact also impact the degree to which policy is informed by research findings [5–8]. The gaping research to policy chasm is exacerbating long-standing health disparities around access to EBP substance use treatment in the USA [9–11].

The USA is in the midst of a more than 10-year opioid epidemic, driven by recent increases in fentanyl-laced drugs and inadequate access to life-saving substance use treatment [12]. In 2021, more than 100,000 people died from an overdose [13]. There is an urgent need to expand access to evidence-based treatment for opioid use disorder—particularly within Medicaid and Children’s Health Insurance Program (CHIP) funded service settings. Medicaid and CHIP provide health insurance benefits for low-income adults and children in the USA, including 38% of individuals in the USA living with an opioid use disorder [9]. Despite a substantial need for care, Medicaid/CHIP members (i.e., individuals who are enrolled in and receive Medicaid/CHIP insurance coverage) have poor access to evidence-based opioid use disorder treatments. Only 48.2% of adult Medicaid [9] and 4.7% of CHIP [14] members living with opioid use disorder receive any evidence-based medications for opioid use disorder (MOUD). Underuse of MOUD is driven by a myriad of misaligned policies and evidence use behaviors across federal, state, and organizational levels that limit MOUD coverage in Medicaid/CHIP benefit arrays and impose non-evidence based utilization management policies that make MOUD difficult to access [15–18]. Improving the use of evidence-informed decision-making in Medicaid/CHIP benefit arrays is critical to expanding access to effective treatments for opioid use disorder and preventing overdoses.

### Policy misalignment and limited access to medications for opioid use disorders in Medicaid

MOUD are first-line, evidence-based treatments for opioid use disorder that include buprenorphine (oral, implantable, injectable), methadone (oral), and naltrexone (oral, injectable) [19]. Buprenorphine and methadone are clinically effective for reducing opioid misuse [20, 21] and overdoses [22–24], and increasing treatment retention [25, 26]. Methadone is not approved for individuals younger than 18 years old, but the American Academy of Pediatrics has strongly endorsed the need to increase access to buprenorphine and naltrexone for youths [27]. Youths with opioid use disorder who receive MOUD have higher rates of treatment engagement than youths receiving behavioral health therapy alone [14]. The National Institute on Drug Abuse (NIDA) has noted that after decades of research demonstrating the efficacy of MOUD, more research on their benefits for substance use treatment is not needed [28]. Instead, NIDA has emphasized the need for research on effective strategies to increase the accessibility and implementation of these medications by overcoming attitudinal barriers [28], including stigma and inconsistent use of evidence-informed policymaking about MOUD across federal and state agencies and payor organizations.

Nationally in the USA, there is momentum for federal policies that support access to MOUD for Medicaid/CHIP members. The Affordable Care Act identified substance use treatment as an essential health benefit and reinforced the Mental Health Parity and Addiction Equity Act requirements to remove benefit limitations on substance use treatment that are more restrictive than limits for medical/surgical benefits. Section 1006(b) of the 2018 federal Substance Use Disorder Prevention that Promotes Opioid Recovery and Treatment for Patients and Communities (SUPPORT) Act requires all Food and Drug Administration approved MOUD be included as mandatory Medicaid state plan benefits [29]. However, the Centers for Medicare and Medicaid Services acknowledges that compliance with parity requirements and SUPPORT Act-mandated MOUD coverage are difficult to enforce and monitor across the 56 state/territory Medicaid/CHIP agencies and more than 250 managed care organizations (MCOs) they contract with to administer benefits [16]. Federal mandates are an important policy lever to increase coverage of MOUD broadly but may be insufficient to increase access to all MOUD formulations or prevent agencies or MCOs from restricting access via utilization management controls.

US state Medicaid/CHIP agencies have flexibility in how they design and implement benefits. Benefit arrays set by Medicaid/CHIP agencies and contracted MCOs can cover MOUD while simultaneously restricting access to certain medications via utilization management policies including non-EBP medical necessity criteria and prior- and re-authorizations for certain medications. For example, 18 state Medicaid agencies require prior authorizations for injectable naltrexone, while 39 agencies require prior authorization for oral buprenorphine [30]. “Fail first” or step therapy policies prevent a provider from initiating treatment with certain MOUD formularies (e.g., injectable) until treatment with other lower cost formularies (e.g., oral) has been unsuccessful [16, 31, 32]. Such practices can increase harms to those with opioid use disorder through increased risk of return to substance use, overdose and death, and conflict with prescriber and client treatment plans [32, 33]. Utilization management policies can promote use of therapeutically superior drugs, but research suggests that many such policies are not evidence-based and serve as treatment barriers [16, 30]. The decentralized administration of Medicaid/CHIP benefits introduces additional opportunities for inconsistent use of evidence-informed policy decisions about MOUD. Most Medicaid/CHIP agencies contract MCOs to administer benefits; nearly 70% of all Medicaid members across the USA are enrolled in MCOs [34]. MCOs can impose their own utilization management restrictions that differ from those of the state agency and are not evidence-based [16, 35]. For example, some MCOs have denied MOUD for members who return to substance use [36]. A recent national survey of Medicaid plan coverage found that nearly 36% of MCOs surveyed require prior authorization for MOUD, with MCOs differentially imposing these policies on access to buprenorphine, methadone, and naltrexone [35].

Identifying the specific influences on and the sources of evidence from which Medicaid/CHIP and MCO policymakers derive guidance when designing benefits is critical to developing strategies that promote evidence-based MOUD coverage in insurance benefit arrays and for reducing health inequities. However, no research exists to describe key determinants of Medicaid/CHIP and MCO policymakers’ decision-making processes or strategies to improve their evidence use behaviors.

### Dissemination science approaches to reduce the “research to policy gap”

Dissemination science offers an interdisciplinary approach to systematically test strategies to improve the translation of research to policy and increase access to healthcare generally and, in particular, MOUD for Medicaid and CHIP members. Dissemination science draws on theory from health services research, political science, public administration, communication, and marketing fields to investigate how EBPs can be optimally communicated to targeted adopters and implementers, such as policymakers, to inform decision-making processes [37, 38]. Dissemination science is useful for investigating and developing active strategies and processes by which policymakers receive, solicit and adopt knowledge about EBPs to make decisions that impact public health [39, 40].

Early policy-focused dissemination research synthesized evidenced about the extent and types of evidence used in policymaking [2, 41, 42]. This work highlighted the need to better understand policymakers’ attitudes and behaviors relevant to the use of research evidence [43]. Purtle et al. made significant contributions documenting state legislators’ and mental health agency officials’ preferences for evidence, including the desirability of data on cost-effectiveness and budget impact when considering behavioral health interventions [44, 45]. Prior research has also characterized state legislators’ prioritization and use of research, suggesting that dissemination strategies should be tailored to specific policymakers to achieve a greater influence their evidence use behaviors [37, 46–49]. Tailored dissemination strategies require considering how to strategically frame the messaging and content of communications about scientific research to increase the odds that such information is timely, easily understandable, persuasive, and useful to policymakers [50]. However, there is insufficient research on both the process for empirically developing tailored dissemination strategies [46, 48], and policymakers’ perceived utility of such strategies.

This study will address these dissemination science knowledge deficits by conducting a US national study of Medicaid/CHIP agency and MCO policymakers’ to empirically identify key determinants, mechanisms, and preferences for evidence use. This study will solicit participation from policymakers in Medicaid/CHIP agencies across all 56 US states and territories and the more than 250 (and growing number of) contracted MCOs. We will use survey results to develop tailored dissemination strategies that promote evidence use when designing MOUD benefits (including utilization management policies) for adult and child members. Evidence use around Medicaid/CHIP MOUD benefits likely differs from evidence use for other populations and behavioral health practices. The historical politicization of Medicaid eligibility [51] and stigma toward individuals living with opioid use disorder or using MOUD are potential outer context determinants that may influence agencies’/MCOs’ compliance with the SUPPORT Act’s MOUD mandate. Leadership within Medicaid/CHIP agencies’ and MCOs’ inner context can also impact decisions about benefits. But the true extent to which contextual factors influence adult and child benefit design is unknown. This limited transparency in Medicaid is often criticized as “black box” policymaking [52–54]. Our study aims to address this longstanding knowledge deficit by explaining the currently abstruse determinants and processes by which Medicaid/CHIP agency and MCO policymakers seek out, receive, and use evidence. A review of the National Institutes of Health RePORTER revealed that this is the first NIDA-funded study examining dissemination strategy development or use. Thus, this study will also generate knowledge about the empirical development and utility of tailored dissemination strategies.

## Methods/design

### Specific aims

This study aims to promote Medicaid/CHIP and MCO policymakers’ use of scientific evidence when designing MOUD benefits. The study aims and hypotheses are described below.

Aim 1: Develop and administer a national survey to Medicaid/CHIP agency and MCO policymakers to identify determinants, mechanisms, and intermediary characteristics that influence their behavior seeking out, receiving, and using research evidence to define MOUD benefits.

Aim 1 Rationale: Although the annual Medicaid Operations Survey collects data about Medicaid activities and priorities [55], these data have major limitations for informing research on policymakers’ evidence use behaviors. Survey responses are from the perspective of Medicaid Directors, do not consider the views of benefit or utilization management policy developers and other staff engaged in benefit decisions, nor do they report on organizational climate. Medicaid Operators Survey data are not publicly available and survey reports describe data in aggregate rather than at the state-level [55]. The present study will create a critically needed dataset to provide transparency about basic components of each agencies’ inner context and their influence over staff evidence use behaviors.

Aim 2: Empirically identify and describe subgroups of Medicaid/CHIP agencies and MCOs with distinct determinants and intermediary relationships that impact their use of evidence in MOUD benefit design.

Hypothesis: Heterogenous classes (comprised of agencies/MCOs) exist with differential evidence use behaviors and intermediaries referenced when designing MOUD benefits for adults and youths.

Aim 2 rationale: Medicaid/CHIP agencies are notorious for differences in structure and benefits for opioid use disorder treatment services [56, 57]. Our national survey developed in aim 1 will highlight additional variations. Latent class analysis (LCA) is finite mixture model approach that moves beyond these differences to classify agencies/MCOs into subgroups (i.e., latent classes) based on their patterns of responses to sets of observed outer and inner context variables and bridging factors [58, 59]. LCA will enable the identification of a manageable number of groups with distinct evidence use behaviors to intervene upon rather than attempting to tailor dissemination strategies to every agency or MCO in the study.

Aim 3: Design and assess the acceptability, appropriateness and feasibility of dissemination strategies, tailored to each latent class, to enhance evidence-informed decision-making for MOUD benefits.

Hypothesis: Tailored dissemination strategies will have higher reported acceptability, appropriateness, and feasibility ratings than strategies that are not tailored to agency/MCO class needs and preferences.

Aim 3 rationale: Dissemination strategies to effectively promote evidence-informed decision-making in Medicaid/CHIP benefits are needed. Prior research suggests that tailored dissemination strategies may be most effective for improving policymakers’ use of research [48, 60], but optimal approaches for tailoring and delivering dissemination strategies is unknown.

We adhered to the Standards for Reporting Implementation Studies to describe study methods (Additional File 1). Study procedures were reviewed and approved by the University of California San Diego Institutional Review Board (Protocol #802208).

### Conceptual framework

This study is guided by the Exploration, Preparation, Implementation, Sustainment (EPIS) Framework, which has demonstrated utility for investigating determinants and mechanisms across the nonlinear dissemination and implementation phases [61–63]. In this project, the *exploration phase* occurs when policymakers in Medicaid and CHIP agencies and MCOs consider the need to change MOUD benefits and marks the first time when dissemination strategies can be first deployed to promote the use of research evidence in decision-making processes. The *preparation phase* describes policymakers’ activities assessing potential barriers and facilitators to rolling out new MOUD benefit policies and when dissemination strategies can be used to effectively communicate Medicaid/CHIP agency benefit decisions with MCOs. During the *implementation phase*, contracted providers deliver MOUD benefits. The *sustainment phase* focuses on continued coverage of evidence-based MOUD benefits over time [64].

**Fig. 1:**
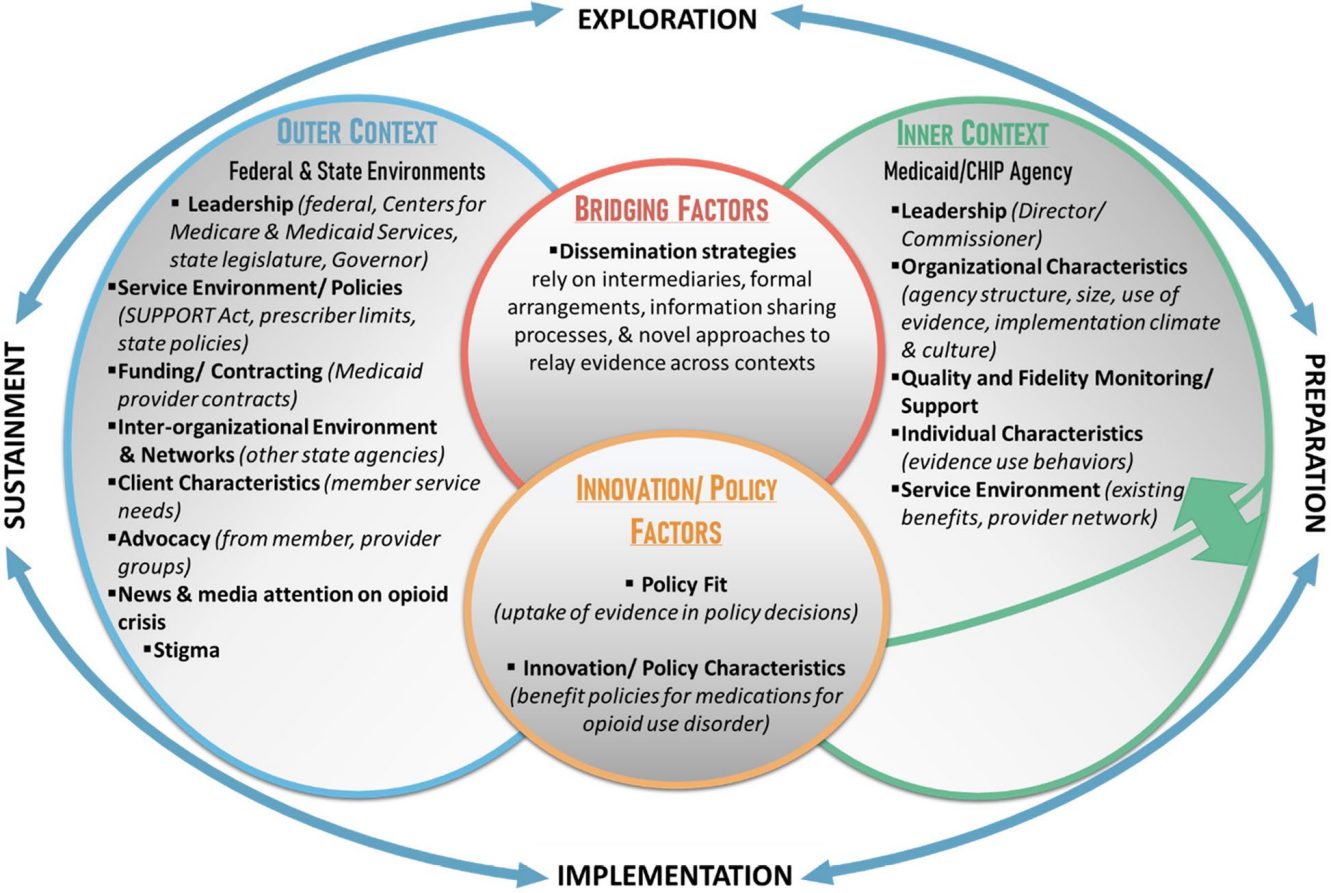
Adapted Exploration, Preparation, Implementation, Sustainment framework for investigating influences on policymaking processes

An adapted EPIS framework (Fig. 1) illustrates how *inner* and *outer contexts* (comprised of potential determinants and mechanisms) and *bridging factors* (i.e., those that link *outer* and *inner contexts*) influence agency and MCO policymakers’ current and preferred processes for receiving and using evidence in policymaking across EPIS phases [65, 66]. The adapted EPIS has a multi-level *inner context*. On one level, it describes the nature of state Medicaid/CHIP agencies’ organizational settings where agency directors can rise through the ranks or can be politically appointed to carry out partisan agendas (e.g., expanding/ limiting benefits). Another level describes the organizational nature of MCOs. Both *inner context* levels consider how agency and MCO leadership, organizational characteristics, service environment (e.g., existing and future MOUD benefits), and quality and fidelity monitoring processes operate and interact to influence policymakers’ engagement in evidence-informed decision-making. The *outer context* includes influences at the federal (e.g., Centers for Medicare and Medicaid Services) and state level (e.g., governor, state legislature who can exert partisan influence) from leadership, state/federal policies (e.g., SUPPORT Act), funding and contracting arrangements that impact the service environment, perceptions/stigma toward Medicaid/CHIP members, and advocacy groups that lobby for or against MOUD. Prior research on opioid use disorder treatment policy highlights the important influence that state legislators and governors have over access to care [6, 67–69], and we expect those *outer context* political entities to similarly influence Medicaid policymaker decisions about MOUD policies.

This adapted EPIS framework considers how *outer context* news and social media attention can influence *inner context* benefit design decisions. This study demonstrates how EPIS can be used to articulate multilevel, cross-context, policy relevant factors in dissemination research [70, 71]. *Bridging factors* [65, 70] acknowledges that intermediaries relay evidence and are critical to dissemination strategy messaging to improve the use of evidence-informed decision-making in MOUD benefit design. *Innovation factors* [70] characterizes the different MOUD benefit policies including which types are included in formularies available to adult and child members. Adapting the EPIS framework in this way will allow us to test its utility for guiding the development of policy-level dissemination science [71].

### Design

Aim 1 will be executed as an observational cross-sectional survey design. Dissemination strategies will be pilot tested in aim 3 using a quasi-experimental design.

### Selection of participants

The study will recruit Medicaid/CHIP agency staff from all 50 states, the District of Columbia, and 5 US territories (American Samoa, Commonwealth of the Northern Mariana Islands, Guam, Puerto Rico, and the US Virgin Islands—whose Medicaid/CHIP benefits are understudied) as well the more than 250 MCOs (the number of MCOs are expected change based on individual state agency contracting agreements) contracted to administer benefits. Eligible participants will include agency/MCO leaders and staff whose work responsibilities include designing or influencing benefits for MOUD. Sample frames will be constructed for the eight staff types with ability to influence Medicaid/CHIP benefit and utilization management policies for MOUD: (1) Medicaid and CHIP agency Directors/Commissioners, (2) MCO executives, (3) Medicaid/CHIP agency and MCO Directors of Behavioral Health, (4) Medicaid/CHIP/MCO Directors of Policy, (5) Medicaid/CHIP/MCO Directors of Budget, (6) Medicaid/CHIP/MCO Healthcare Benefit Developers, (7) Medicaid/CHIP/MCO Pharmacy Benefit Developers, and (8) Medicaid/CHIP/MCO Utilization Management Policy Staff.

### Methods

#### Aim 1 survey instrument and measures

A cross-sectional web-based survey will be administered to Medicaid/CHIP agency and MCO staff (i.e., MOUD benefit policymakers) to collect data on agency-level decision-making. Survey items will map to EPIS constructs to illuminate influences across outer and inner contexts. Survey items draw from existing reliable scales like the Implementation Leadership Scale [72], Purtle et al.’s Research Dissemination Barriers survey items [37], and the Six Factor Model of Evidence-based Decision-making Tool [73] to assess policymakers’ behaviors seeking, receiving, and using research. Survey items will inquire separately about the use of research when developing adult and youth MOUD benefits. Few scales assess outer context policy determinants [74], so we will develop items to collect data about these factors (e.g., need for legislative approval to alter benefits, partisanship, stigma) and intermediaries who share research. To reduce respondent burden, items quantifying MOUD benefit policies and state policy landscapes will be pre-filled using publicly available information from agency/MCO websites, contracts, and other policy documents.

#### Cognitive pre-testing of the policymaker survey

Several of the survey items have been used in prior studies examining legislators’ evidence preferences and behaviors. However, these items have not been tested with Medicaid/CHIP agency or MCO staff. To promote instrument relevance, clarity, and brevity, we will cognitively pre-test the survey with at least five different policymakers from Medicaid/CHIP agencies and/or MCOs before fielding the national survey. The cognitive pre-testing process will include conducting a phone or video call interview to verbally deliver the survey items and ask questions about each. Participants will be asked to think aloud about survey items, describe how they comprehend each item, retrieve information to respond, how confident they feel responding to specific items, and offer general impressions about the instrument [75]. Participants will also be provided with a web-link to interact with the web-based survey during the interview and report on the user-friendliness of its interface. We will revise the survey instrument to be responsive to participant feedback.

#### Survey recruitment and data collection

Eligible Medicaid/CHIP agency and MCO policymakers will receive an email containing a brief study description and invitation to participate in the Qualtrics online survey. Conservatively, we estimate there are at least 2261 eligible respondents from the 56 agencies and 267 MCOs (the number of MCOs is subject to change based on contract arrangements in each state; there were 267 contracted MCOs at the time of publication). This approximation of eligible respondents is based on estimating that each agency/MCO includes one staff member working in each of the following eight key informant roles: Medicaid/CHIP Directors or Commissioners, MCO executives, Behavioral Health Director, Policy Director and Budget Director, Healthcare Benefit Developer, Pharmacy Benefit Developer, and Utilization Management Policy Staff whose purviews include MOUD. Directors/executives will be permitted to designate a staff member to complete the survey on their behalf. Prior survey work by Grogan et al. achieved a 92% Medicaid agency response rate [56], while other surveys suggest 35–45% is a realistic response rate from policymakers [76, 77]. We anticipate achieving at least a 35% response rate (*N* = 791) using recruitment strategies that have been successful in prior surveys with policymakers, including the development of data frames with contact information for each stakeholder type, and by introducing the study at relevant conferences where these policymakers convene [6, 56, 67, 76].

Eligible policymakers will receive an email containing a brief study description and the online survey link. This email will also include the opportunity identify additional eligible policymakers via snowball sampling methods. Email messages will include a request for the recipient to provide contact information for other potentially eligible staff in their agency/MCO. They will be able to provide this contact information via email and/or by submitting the contact information via a separate weblink embedded within the email. This snowball sampling recruitment method will allow the research team to review the contact information and assess each referred staff members’ eligibility before emailing them the study description and online survey invitation. Eligible policymakers will receive up to 10 emails asking them to complete the linked survey. After the fourth email, we will conduct up to eight calls with policymakers to ensure they received the survey link, answer their questions, and encourage them to complete the survey. We will not compensate participants since state employees cannot accept such payments.

### Descriptive analysis of survey data (aim 1)

Benefit policies are developed with input from Medicaid/CHIP agency and MCO leaders who set organizational priorities and the mid-level staff who use their own knowledge to draft policy language [78]. Thus, we consider Medicaid/CHIP agencies and MCOs as “actors” whose behavior is the sum of leaders’ and staff knowledge and preferences [79]. We will aggregate individual-level survey responses within each agency/MCO to the organizational level. Using agencies/MCOs as the unit of analysis for survey data will facilitate the development of dissemination strategies tailored to agency/MCO behavior rather than the individual needs of different staff members with varying roles, responsibilities, and influence. This will also prevent the deployment of different dissemination strategy messages to colleagues in the same agency/MCO in aim 3. To determine if survey results are driven by certain inner/outer context characteristics specific to the sample, we will calculate and apply non-response adjusted weights to account for factors like Medicaid expansion status, region, partisanship, separate vs. combined Medicaid/CHIP agency structures, current MOUD benefits, and respondent type using a sample post-stratification approach [80]. Descriptive statistics from aggregated survey data will describe agency/MCO characteristics, intermediary types, and evidence-informed decision-making behaviors when designing adult and youth MOUD benefit policies.

### Latent class analysis of survey data (aim 2)

Medicaid/CHIP agencies are notorious for differences in structure and benefits for opioid use disorder treatment services [56, 57]. Our national survey will highlight additional variations. Latent class analysis (LCA) is finite mixture model approach that moves beyond these differences to classify agencies/MCOs into subgroups (i.e., latent classes) based on their patterns of responses to sets of observed inner/outer context and bridging factor variables [58, 59]. LCA maximizes homogeneity within each identified class so that agencies/MCOs grouped together are as similar as possible, while also maximizing heterogeneity between classes to ensure that classes are mutually exclusive [58, 81].

A preliminary review of the literature and research team expertise in Medicaid policy was used to identify key variables from the outer context (i.e., need for legislative approval, state partisanship) and inner context (i.e., agency structure, evidence-based decision-making behaviors/preferences, implementation leadership), and intermediary types that will serve as indicators of latent classes. Given the exploratory nature of this aim, we will consider additional indicators as data are collected and analyzed.

#### LCA approach

The latent class analysis will be conducted using data collected from individuals representing all respondent agencies and MCOs. Latent classes are best identified through a combination of statistical fit and conceptual interpretation [58, 59]. A frequency distribution of the most common evidence-informed decision-making behaviors/preferences observed in the survey data will be used to manually estimate the expected number of classes for adult and youth benefit design approaches. To determine the optimal number of classes, we will separately test multiple class solutions (e.g., 1-class, 2-class…5 class) for adult and youth MOUD benefits design. Model fit will be assessed using fit indices: Akaike Information Criterion, Bayesian Information Criteria [81], bootstrap Lo-Mendell-Rubin test [82], and entropy (the percentage of agencies/MCOs in the sample that were correctly classified given the specific class model) [59]. We will also consider how interpretable classes are and the model’s compatibility with the initial manual estimate.

Power calculations for LCA models are underdeveloped and unreliable [83, 84]. However, our projected sample is sufficient to identify models that adequately describe the data [85]. Using the best fit models (1 for adult, 1 for youth benefits), we will investigate evidence-informed decision-making item-response parameters to descriptively label each models’ latent classes and determine whether classes in adult and youth models are similar. We will use multinomial logistic regression to evaluate predictors of class membership. Predictive models will be formally evaluated using the 3-step approach [86]. This method simultaneously estimates the best-fitting LCA solution while evaluating the associations between class membership and predictor variables, thus accounting for the uncertainty of class membership [86–88]. Empirically grouping agencies/MCOs allows for unanticipated classes to emerge using observed determinants of evidence use, rather than imposing researcher assumptions about potential determinants.

Validation of LCA results will also depend on the proportion of the entire study sample that best fits into each identified latent class. For example, if one of the classes identified is very small, we will compare descriptive statistics for each class to identify meaningful differences between them as well as the conceptual underpinnings of each class. Descriptive summaries of each class will be presented to a small group of policymakers (e.g., individuals who participated in cognitive pre-testing of the national survey) to solicit their insights on the results. Their feedback will help determine if the identified latent classes really represent the different types of policymakers they have worked with throughout their careers at different Medicaid/CHIP agencies and MCOs.

There are some known differences in state Medicaid policies and such differences suggest that it is likely that two or more relatively distinct latent classes could be identified. However, if meaningfully distinct latent classes of evidence use profiles for adult Medicaid and/or adolescent CHIP MOUD benefit are not found, such a result would be an insightful finding in and of itself. For example, a lack of distinct latent classes would indicate that evidence dissemination strategies could be developed for broad audiences of policymakers rather than needing to develop multiple tailored strategies for different groups of policymakers. Then, the outcomes of such strategies could be examined across multiple states. Thus, a finding of distinct latent classes or no latent classes will provide meaningful insights into evidence use behaviors for Medicaid/CHIP policymakers.

### Dissemination strategy development methods (aim 3)

Using latent class predictors identified in aim 2, we will design adult (i.e., Medicaid) and child (i.e., CHIP) class-specific policy dissemination strategies with the common aims of (1) facilitating evidence exchange between agency and MCO policymakers and research-driven intermediaries and (2) promoting the uptake of evidence-based MOUD benefits. Thus, dissemination strategies will serve as new bridging factors to share evidence between outer context entities and inner context policymakers [66].

Dissemination strategies require clearly defined sources, messages, channels, and audiences [89, 90] and bridging factors research recommends specifying function (i.e., purpose) and form (i.e., how it is tailored to local context) [66]. We will use a 10-step process (Table 1) to develop and specify strategies. In step 1, we will identify a statistically significant class predictor from aim 2 (e.g., medication cost is a determinant in MOUD benefit decisions) that will serve as the dissemination strategy’s message target. Then, we will review literature to assess whether the message target is aligned with EBP MOUD treatment (step 2). If it is misaligned with evidence, a dissemination strategy will be developed with data-informed functions and forms (steps 3-10). This 10 step process aligns with best practices for strategy specification [91] and will advance methods for tailoring dissemination strategies to serve as policy implementation bridging factors.

**Table 1:** Ten-step tailored dissemination strategy development process

	Steps for developing tailored dissemination strategies		Example dissemination strategy
**Function defining steps:** *characteristics that define the dissemination strategy’s purpose*	**Step 1**	Identify message target (i.e., the determinant, mechanism, or intermediary influencing evidence use)	Benefit design choices are driven by costs: only lower cost medication for opioid use disorder types (e.g., oral) are included on the preferred drug list. More expensive types (e.g., injectable) are excluded
	**Step 2**	Review literature to assess if the message target is aligned with evidence-based practices	Determinant is misaligned with prescribing guidelines that emphasize patient need over cost
	**Step 3**	Specify the intended dissemination outcome (i.e., benefit policy change)	Adoption of all medication for opioid use disorder types on preferred drug list (not just least expensive types)
	**Step 4**	Justify the intended outcomes using current medical guidelines and other research evidence sources	Prescribing a medication for opioid use disorder type (e.g., oral vs. injectable) should be a decision between the prescriber and patient, based on the patient medical history and patient preferences
	**Step 5**	Identify the preferred source (intermediary) to deliver the research evidence	Economist affiliated with local university (*assumes agency is in a class that prefers to receive evidence from university)
	**Step 6**	Develop a data-driven, evidence-informed message	Data describing costs of treatment failure (e.g., costs of emergency department visits) when patients' medication for opioid use disorder needs and preferences are not met
	**Step 7**	Specify which audience will receive dissemination strategy message	Health and pharmacy benefit developers, agency directors and managed care organization executives
**Form defining steps:** *activities that tailor the dissemination strategy to local needs*	**Step 8**	Identify the preferred communication channel	Agency/organization prefers research summaries and issue briefs
	**Step 9**	Identify the best timing for deploying strategy	Prior to annual medications for opioid use disorder benefits review
	**Step 10**	Identify the appropriate messaging dose	Multiple time points in different venues where leaders convene

### Measuring of acceptability, appropriateness, and feasibility of tailored dissemination strategies with policymakers

We will identify one agency/MCO from each latent class to participate in the pilot study testing tailored dissemination strategies. At least two latent classes are expected to be identified in aim 2. We expect that fourteen individuals from agencies/MCOs represented in each of those classes will participate in the pilot study. Each participating agency/MCO will be assigned strategies tailored to their class (i.e., matched) and strategies tailored to the other classes (i.e., mismatched). Participants will complete a brief survey evaluating the acceptability (satisfaction with content/delivery), appropriateness (relevance, usefulness of evidence), and feasibility (practicability for using presented evidence when designing MOUD benefits) [92] of each tailored dissemination strategy. Survey items derive from Weiner et al.’s four-item measures of implementation outcomes [93]. These measures are widely used in dissemination and implementation science research and may be used independently or together. These measures of acceptability (*α* = 0.85, 4 items), appropriateness (*α* = 0.91, 4 items), and feasibility (*α* = 0.89, 4 items) have demonstrated discriminant content validity and structural validity [93].

### Analysis of pilot study data

Although inferential statistics are not appropriate for pilot studies [94–96], group sample sizes of 14 and 14 (assumes ≥ 2 classes are detected) would achieve 82.4% power to reject the null hypothesis of equal means when the population mean different reflects a Cohen’s *d* = 1.00. Descriptive statistics will characterize the overall perceptions of each dissemination strategy and we will explore between agency differences and between stakeholder type differences in strategy ratings.

## Discussion

### Innovation and impact

This study will illuminate key determinants, mechanisms, and intermediaries influencing policymakers’ evidence-based decision-making behaviors and preferences when designing benefits for MOUD, shedding light on the “black box” policymaking processes in Medicaid/CHIP benefit arrays [52–54]. This study will also produce a critically needed set of data-driven policy dissemination strategies tailored to the specific evidence use behaviors and preferences of Medicaid/CHIP agencies and their contracted MCOs. Although latent class methods are usually conducted with individual-level data [97], our novel application in aim 2 will analyze data at the Medicaid/CHIP and MCO level to reveal organizational-level subgroups. This innovative, empirical approach allows for unanticipated subgroups to emerge based on key determinants of evidence use, rather than imposing researcher-driven assumptions about which factors are more or less important to target. This process allows for the development of tailored dissemination strategies that address the strongest predictors of evidence use for each subgroup of policymakers. Study findings will advance a nascent body of research examining characteristics of policymakers who are receptive to tailored dissemination strategies on different health topics [46, 48, 49].

The methodology proposed in this study will also yield generalizable findings about Medicaid/CHIP agencies and MCOs on a national level. This is critical since the external validity of many Medicaid studies is limited due to heterogeneity in agency structures and policies [56, 57]. Study findings will provide a roadmap for empirical research developing and testing the effect of tailored dissemination strategies on policymakers’ use of evidence when designing policies impacting substance use treatment.

### Considerations and limitations

Survey methods introduce the potential for non-response bias if individuals who do not respond to the survey differ in meaningful ways from those who do participate. To reduce nonresponse, the research team will make several attempts to solicit participation and secure survey responses. We have already conducted preliminary informational interviews with multiple Medicaid policymakers to discuss the best approaches to introduce the study survey and generate participation from their peers; these suggestions will inform the development of all outreach materials. To address nonresponse issues, we will create a category for each variable and attempt to model non-response/missing data as an additional category of indicators used in the LCA. This will enable us to explicitly model missing data as a category for each indicator rather than having the LCA simply “ignore” any missing variables.

Self-reported survey data can also invite recall and social desirability biases. However, these survey methods are established, practical approaches for obtaining data on policymakers’ evidence-informed decision-making behaviors [67, 98]. Although qualitative interviews could provide more in-depth descriptions of policymakers’ evidence use behaviors and preferences these methods are not practical given the study goals. It would not be feasible to conduct, analyze, and synthesize data from individual interviews with participants across the 56 Medicaid/CHIP agencies and 267 MCOs. However, to promote internal validity of survey data, the research team will conduct follow-up calls with individuals who provide incomplete or unclear survey responses.

### Dissemination plans

It is important to note that the SUPPORT Act requirement for Medicaid agencies to include all MOUD in their benefit arrays is set to expire in 2025 [29]. It is unclear what impact the existing provision has had to date and if that requirement will be reinstated beyond 2025. Regardless, dissemination strategies that effectively promote more evidence-informed Medicaid/CHIP benefits will be necessary to reduce disparities in MOUD access. If the requirement to include all MOUD in Medicaid benefits is extended, agencies may be eager to learn about effective dissemination strategies and implementation plans from states participating in this study. Study findings will be disseminated in a variety of formats (e.g., infographics, informational briefs) and venues (e.g., webinars, conferences) to ensure widespread knowledge sharing.

Research on effectively translating scientific evidence into policy is necessary to resolve long-standing health disparities, including in accessing high quality substance use treatment. It is not enough for researchers to publish scientific findings in peer reviewed journals; new strategies for communicating evidence with policymakers are needed to bridge the research to policy gap. This study offers an opportunity to generate practical dissemination strategies that are responsive to policymaker needs, as opposed to designing strategies that are designed for theoretical impact [99]. The results from this study will provide a deeper understanding of how policymakers across state and payor organizations use and prefer to receive evidence to inform health policy and which types of dissemination strategies have the greatest utility for policymakers.

## Supplementary Information

**Additional file 1.**

## Data Availability

Not applicable.

## Abbreviations

EBP: Evidence-based practice
EPIS: Exploration, Preparation, Implementation, Sustainment Framework
CHIP: Children’s Health Insurance Program
LCA: Latent class analysis
MCO: Managed care organization
MOUD: Medications for opioid use disorder
NIDA: National Institute on Drug Abuse
SUPPORT Act: Substance Use Disorder Prevention that Promotes Opioid Recovery and Treatment for Patients and Communities Act
