# Correction: Tailoring dissemination strategies to increase evidence-informed policymaking for opioid use disorder treatment: study protocol

**DOI:** 10.1186/s43058-023-00406-6

**Published:** 2023-03-17

**Authors:** Erika L. Crable, Colleen M. Grogan, Jonathan Purtle, Scott C. Roesch, Gregory A. Aarons

**Affiliations:** 1 Department of Psychiatry, University of California, San Diego, La Jolla, CA USA; 2 Child and Adolescent Services Research Center, San Diego, CA USA; 3 University of California, San Diego Altman Clinical and Translational Research Institute Dissemination and Implementation Science Center, La Jolla, CA USA; 4 Crown Family School of Social Work, Policy, and Practice, The University of Chicago, Chicago, IL USA; 5 Department of Public Health Policy and Management, New York University School of Global Public Health, New York City, NY USA; 6 Global Center for Implementation Science, New York University School of Global Public Health, New York City, NY USA; 7 Department of Psychology, San Diego State University, San Diego, CA USA

**Correction: Implement Sci Commun 4, 16 (2023)**

**https://doi.org/10.1186/s43058-023-00396-5**

Following publication of the original article [1], the authors identified an error in Fig. 1. The correct figure is given below.

**Fig. 1:**
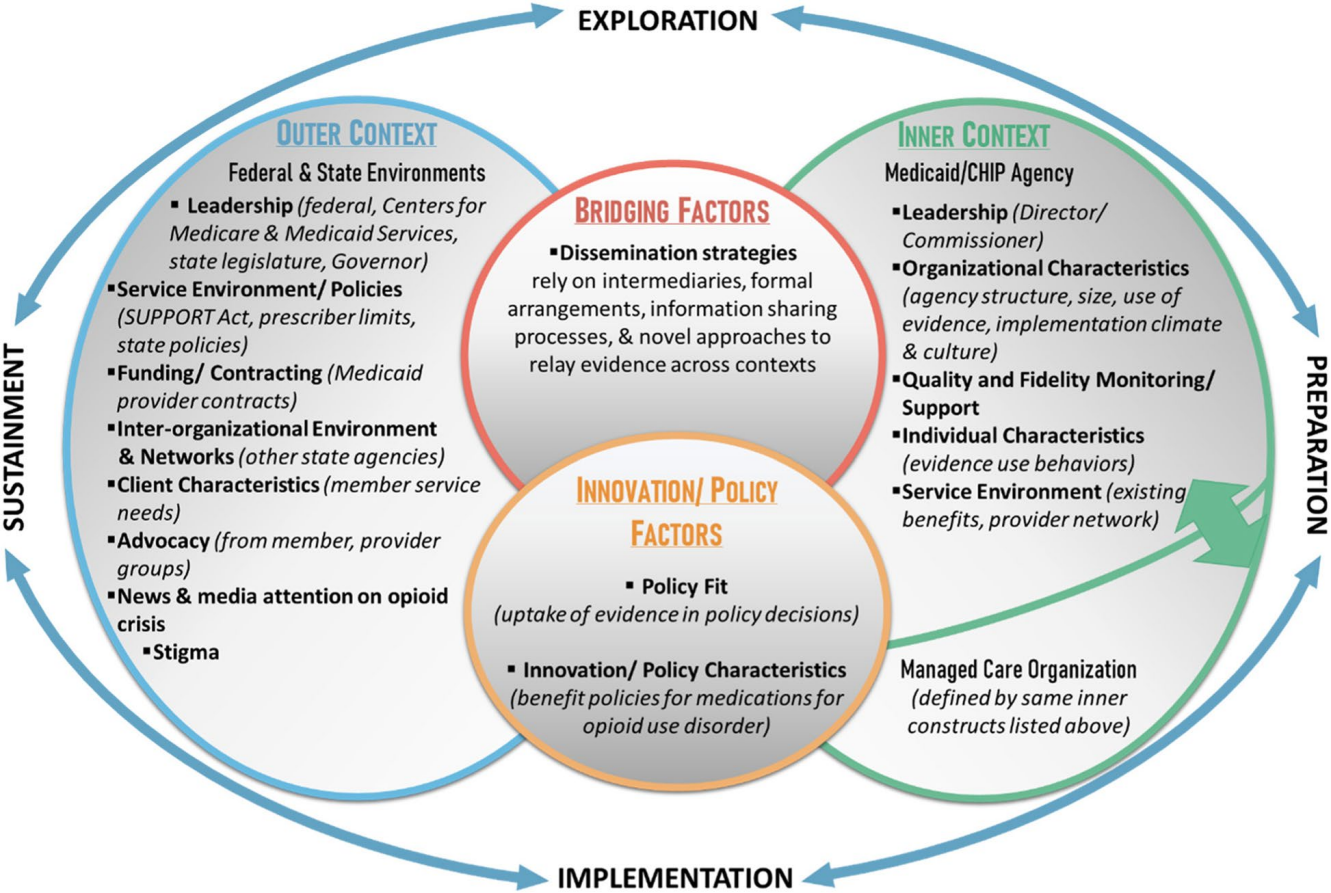
Adapted Exploration, Preparation, Implementation, Sustainment framework for investigating influences on policymaking processes
